# Circulation of Nor98 Atypical Scrapie in Portuguese Sheep Confirmed by Transmission of Isolates into Transgenic Ovine ARQ-PrP Mice

**DOI:** 10.3390/ijms221910441

**Published:** 2021-09-28

**Authors:** Mafalda Casanova, Carla Machado, Paula Tavares, João Silva, Christine Fast, Anne Balkema-Buschmann, Martin H. Groschup, Leonor Orge

**Affiliations:** 1 Histopathology Facility, Instituto Gulbenkian de Ciência (IGC), 2780-156 Oeiras, Portugal; mccasanova@igc.gulbenkian.pt; 2 Veterinary Medicine Department, University of Évora, 7004-516 Évora, Portugal; 3 Pathology Laboratory, UEISPSA, National Institute for Agricultural and Veterinary Research (INIAV), I.P., 2780-157 Oeiras, Portugal; carlitaneves@gmail.com (C.M.); joao.silva@iniav.pt (J.S.); 4 Pathology Laboratory, UEISPSA, National Institute for Agricultural and Veterinary Research (INIAV), I.P., 4485-655 Vairão-Vila do Conde, Portugal; paula.tavares@iniav.pt; 5 Institute of Novel and Emerging Infectious Diseases, Friedrich-Loeffler-Institut, Insel Riems, 17493 Greifswald, Germany; Christine.Fast@fli.de (C.F.); Anne.Balkema-Buschmann@fli.de (A.B.-B.); martin.groschup@fli.de (M.H.G.); 6 Animal and Veterinary Research Centre (CECAV), Associate Laboratory for Animal and Veterinary Science—AL4AnimalS, University of Trás-os-Montes and Alto Douro (UTAD), 5000-801 Vila Real, Portugal

**Keywords:** prion, transmissible spongiform encephalopathies, atypical scrapie, transgenic mice

## Abstract

Portugal was among the first European countries to report cases of Atypical Scrapie (ASc), the dominant form of Transmissible Spongiform Encephalopathy (TSE) in Portuguese small ruminants. Although the diagnostic phenotypes observed in Portuguese ASc cases seem identical to those described for Nor98, unequivocal identification requires TSE strain-typing using murine bioassays. In this regard, we initiated characterization of ASc isolates from sheep either homozygous for the ARQ genotype or the classical scrapie-resistant ARR genotype. Isolates from such genotypes were transmitted to TgshpXI mice expressing ovine PrPARQ. Mean incubation periods were 414 ± 58 and 483 ± 107 days in mice inoculated with AL_141_RQ/AF_141_RQ and AL_141_RR/AL_141_RR sheep isolates, respectively. Both isolates produced lesion profiles similar to French ASc Nor98 ‘discordant cases’, where vacuolation was observed in the hippocampus (G6), cerebral cortex at the thalamus (G8) level, cerebellar white matter (W1) and cerebral peduncles (W3). Immunohistochemical PrP^Sc^ deposition was observed in the hippocampus, cerebellar cortex, cerebellar white matter and cerebral peduncles in the form of aggregates and fine granules. These findings were consistent with previously reported cases of ASc Nor98 transmitted to transgenic TgshpXI mice, confirming that the ASc strain present in Portuguese sheep corresponds to ASc Nor98.

## 1. Introduction

Scrapie belongs to a group of diseases called transmissible spongiform encephalopathies (TSEs) or prion diseases. TSEs are caused by conversion of a natural prion protein (PrP^C^) into an abnormal prion protein (PrP^Sc^), which accumulates in affected tissues of the infected host, leading to neural degeneration [1]. While PrP^C^ is completely hydrolyzed by proteinase K, PrP^Sc^ has a proteinase K-resistant core (PrP^res^), constituting the diagnostic hallmark of transmissible prion diseases. Prion differentiation is performed through evaluation of detergent solubility, as well as biochemical properties and electromobility of PrP^res^ [2,3].

TSEs include Creutzfeldt-Jakob disease (CJD) in humans, Bovine Spongiform Encephalopathy (BSE) in cattle, Scrapie in small ruminants, Feline Spongiform Encephalopathy (FSE) in felids, and Chronic Wasting Disease (CWD) in cervids [4,5]. Currently, there are two known forms of scrapie infecting small ruminants, classical scrapie (CSc) and atypical scrapie (ASc). CSc is a transmissible form of scrapie that was first diagnosed nearly 300 years ago [6]. Sheep susceptibility to CSc is determined by polymorphisms in the prion protein gene (prnp), particularly at codons 136, 154 and 171. V136R154Q171 and A136R154Q171 sheep are the most susceptible genotypes, whereas A136R154R171 and A136H154Q171 are associated with relative resistance to the disease [7]. ASc has been reported in older sheep and in sheep with the prnp CSc-resistant AHQ and ARR alleles, as well as the AF141RQ allele [8,9,10]. ASc was first diagnosed in 1998 in Norway, but there is evidence it has existed since as early as 1972 [11]. Unlike CSc, outbreaks of ASc appear to be spontaneous. In addition, ASc tends to disseminate poorly within a flock [1,12].

The European Union active scrapie surveillance plan was implemented in 2002 following the European BSE crisis [13]. This program consists of testing a representative number of healthy slaughtered and fallen stock animals older than 18 months (active surveillance). Animals with suspected clinical signs of disease must also be tested (passive surveillance). Testing involves collection of brainstem samples, at the level of the obex, to be tested by an EU wide approved TSE rapid test. All positive results must be submitted for confirmatory tests, including Western immunoblotting, histopathology or immunohistochemistry (IHC). Additionally, genotyping must be performed [14].

ASc was first diagnosed in Portuguese sheep in 2003. The first seven cases were identified following the EU active surveillance plan, leading to testing of approximately 30,000 small ruminants for TSEs [15]. Unlike other European countries, ASc is the dominant form of scrapie in Portuguese sheep, and CSc was only identified, for the first time, in 2008. Until the end of 2020, Portugal reported 779 ASc cases (713 Portuguese sheep, 45 imported sheep; 15 Portuguese goats and 6 imported goats) and 45 CSc cases (39 Portuguese sheep and 6 imported sheep) in small ruminants.

Following confirmation of the first Portuguese ASc cases, this study aimed to strain-type the disease using murine bioassays. Two brainstem isolates from sheep belonging to AL_141_RQ/AF_141_RQ and AL_141_RR/AL_141_RR genotypes were selected for this study. Each isolate was transmitted to a cohort of transgenic ovine mice expressing ovine PrPARQ (TgshpXI).

## 2. Results

Four out of the initial 30 animals died less than 100 days post-inoculation and were excluded from analysis. Three of these mice had been inoculated with the ARR isolate and one mouse inoculated with the ARQ isolate. This resulted in a total of 26 brains submitted for Western immunoblot analysis, 14 mice of AL_141_RQ/AF_141_RQ genotype and 12 mice of AL_141_RR/AL_141_RR genotype. Of the 14 mice analyzed after challenge with the ARQ isolate, 10 were confirmed PrP^Sc^ positive, while all 13 mice inoculated with the ARR isolate were PrP^Sc^ positive. Mean incubation periods of these positive mice were 418 ± 55 days for AL_141_RQ/AF_141_RQ cohort and 483 ± 102 days in AL_141_RR/AL_141_RR cohort.

**Figure 1:**
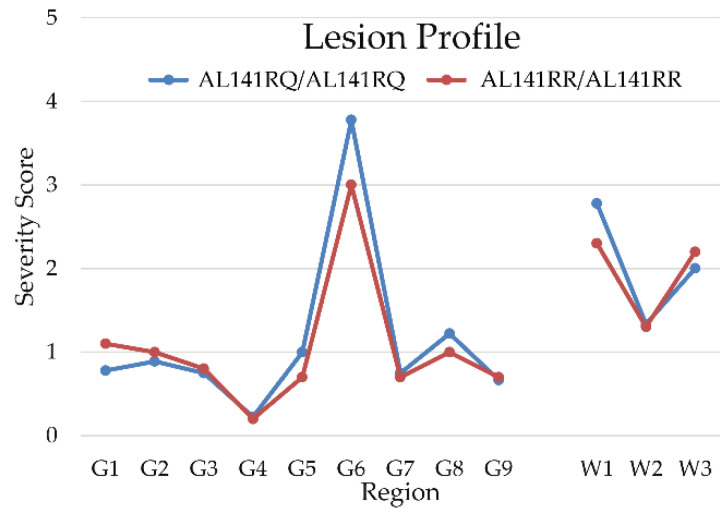
Mean vacuolation severity scores of lesion profiles of transgenic TgshpXI mice inoculated with Portuguese atypical scrapie of nine grey matter (G) and three white matter (W) regions, showing peaks at hippocampus, cerebellar white matter and peduncles. The blue and red lines describe the mean severity score of vacuolation produced by transmission of cases from AL_141_RQ/AF_141_RQ and AL_141_RR/AL_141_RR genotypes, respectively. Abbreviations: G1, dorsal medulla; G2, cerebellar cortex; G3, superior colliculus; G4, hypothalamus; G5, thalamus; G6, hippocampus; G7, septum; G8, medial area of the cerebral cortex at the level of the thalamus; G9, medial area of the cerebral cortex at the level of the septum; W1, cerebellar white matter; W2, decussation; W3, cerebral peduncles.

**Figure 2:**
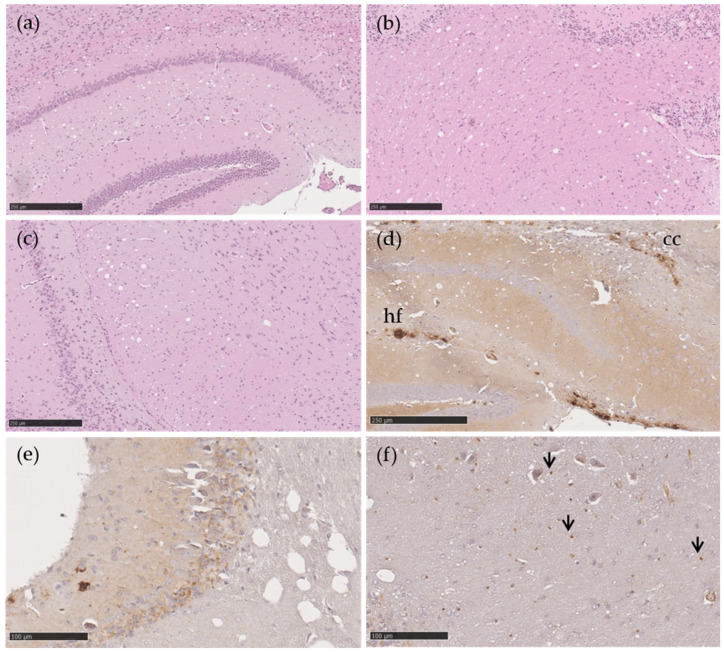
Vacuolation and PrP^Sc^ in transgenic TgshpXI mice inoculated with Portuguese atypical scrapie AL_141_RR/AL_141_RR (**a**–**d**) and AL_141_RQ/AF_141_RQ (**e**,**f**) sheep isolates. Moderate vacuolation severity at the level of the hippocampus (**a**), cerebellar white matter (**b**) and cerebral peduncles (**c**). Aggregates and fine granular PrP^Sc^ types in the hippocampus and corpus callosum (**d**) and in the molecular layer of the cerebellum (**e**). Punctate PrP^Sc^ (arrow) in the white matter of the cerebellum (**f**). H&E (**a**–**c**), monoclonal 2G11 antibody (**d**–**f**). Scale bar = 250 µm (**a**–**d**), 100 µm (**e**,**f**). Abbreviations: cc, corpus callosum, hf, hippocampal fissure.

Lesion profiles of TgshpXI mice inoculated with both genotypes are provided in Figure 1. The blue line refers to mice inoculated with AL_141_RQ/AF_141_RQ genotype and the red line refers to mice inoculated with AL_141_RR/AL_141_RR genotype. Both groups of mice exhibited vacuolation in the hippocampus (G6), cerebral cortex at the level of the thalamus (G8), cerebellar white matter (W1) and cerebral peduncles (W3) (Figure 2a–c). However, in comparison with the AL_141_RR/AL_141_RR genotype, mice belonging to the AL_141_RQ/AF_141_RQ cohort demonstrated overall increased severity in their lesions, despite shorter incubation periods.

The immunohistochemical features of both isolates revealed the same PrP^Sc^ deposition patterns. PrP^Sc^ deposition was observed in the hippocampal fissure and corpus callosum in the form of moderate aggregates and fine granular PrP^Sc^ structures (Figure 2d), in the cerebellum (molecular layer) as mild fine granules (Figure 2e), as well as in the cerebellar white matter in the form of punctuate (Figure 2f).

**Figure 3:**
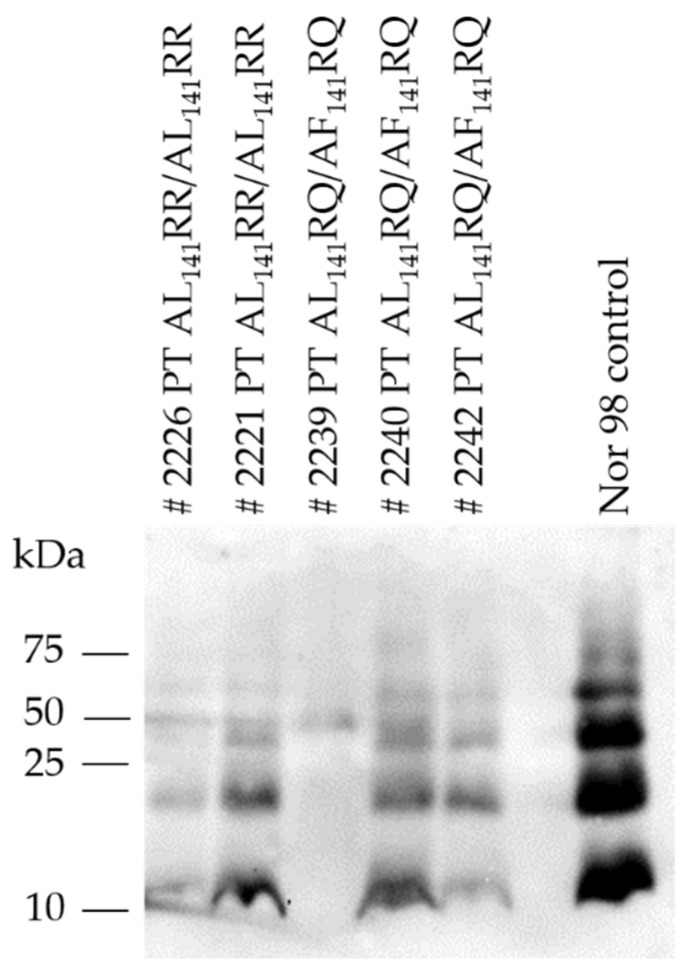
Western immunoblot of PrP^Sc^ from brains of transgenic TgshpXI mice inoculated with Portuguese (PT) atypical scrapie isolates (# indicating mouse ID). Nor98 atypical scrapie was included as a control and displayed the characteristic atypical prion protein banding pattern with 4–5 bands of fragment sizes 12–60 kDa.

Western immunoblot analysis of brains of TgshpXI showed the typical multi-band pattern of immunoreactive bands of PrP^Sc^ between 12–60 kDa with the prominent low molecular mass band of approximately 12 kDa (Figure 3).

## 3. Discussion

The results of this study indicate the ASc strain present in Portuguese sheep is indistinguishable from ASc Nor98. Both Portuguese isolates (from sheep of AL_141_RR/AL_141_RR and AL_141_RQ/AF_141_RQ genotypes) caused vacuolation peaks in regions G6 (hippocampus), G8 (cerebral cortex at the level of the thalamus) and W3 (cerebral peduncles). These findings coincided with the characteristic lesion profiles observed in other transgenic PrP mouse models inoculated with atypical scrapie [2,11,16,17]. Additionally, there was severe vacuolation in the cerebellar white matter (W1), as previously described for Tg338 mice inoculated with ASc French ‘discordant’ cases [2,17]. Previous studies using mouse bioassays showed similar Western immunoblot mobility patterns, but also having slight variations in lesion profiles, incubation periods, and PrP^Sc^ deposition, depending on the mouse line used for determination of lesion profiles. Collectively, these findings suggest ASc is caused by a single prion strain [18].

The isolate from the AL_141_RQ/AF_141_RQ genotype promoted an increase in lesion severity with shorter incubation periods. Similarly, a previous study found inoculation of isolates from donors with prnp alleles linked to higher susceptibility to ASc lead to shorter incubation periods for lesion development in transgenic TgOvPrP4 mice, in comparison with those belonging to sheep with prnp alleles associated with ASc resistance [19]. Notably, in this context, is having the ARQ/ARQ genetic background in transgenic Tgshp XI mice renders them more susceptible to develop disease with the AL_141_RQ/AF_141_RQ isolate.

Additionally, the PrP^Sc^ profile showing marked deposition in the hippocampal fissure, corpus callosum, cerebellum, and cerebellar white matter, is similar to those reported cases of ASc transmitted to transgenic TgshpXI mice [2].

Both ASc affected sheep were diagnosed after observation of discrete vacuolation of the spinal tract nucleus of the trigeminal nerve, in addition to globular PrP^Sc^ deposits in the white matter at the level of the obex. Unfortunately, in archived sheep tissues examined for this study, collection of the cerebellum was not a standard procedure at that time; thus, it was not possible to verify the PrP^Sc^ deposition in this region. The variability in PrP^Sc^ neuroanatomical distribution in the natural host is well-known [9,20]. However, the impact of such variations on the lesion and PrP^Sc^ profile in transgenic mice remains uncertain. Hence, it is important to study the influence of such variations in murine bioassays, by selecting cases with different neuroanatomical distribution, in order to ascertain presence of differing atypical scrapie strains, as well as other factors, which may determine such variability.

Recent work revealed the possibility of the development of a classical-BSE (BSE-C) prion after inoculation of bovine PrP transgenic mice with ASc isolates [3]. That study found that BSE-C may be present in natural ASc isolates as a minor variant, and transmission of such isolates to transgenic bovine mice resulted in emergence of BSE-C as a dominant variant. The same phenomenon was not observed after inoculation of CSc isolates. Hence, there is concern regarding the possibility of ASc having a role in the emergence of BSE-C in cattle, and a possible role in the origin of the 1980s BSE crisis, resulting from inclusion of rendered small ruminants in cattle feed [3]. Furthermore, archival ASc isolates reveal ASc was present in the United Kingdom years before BSE [11]. Another study found oral transmission of ASc into sheep has resulted in a phenotype shift to CH1641, a classical scrapie strain showing an immunoblot profile similar to bovine BSE. Although CH1641 has not been diagnosed in Portuguese sheep as of yet, it is prudent to maintain vigilant systematic analysis of lesion profiles, PrP^Sc^ immunolabelling types and patterns, as well as PrP^Sc^ electrophoretic profiles in natural hosts for evidence of any phenotypic shift and strain conversion. Such surveillance is particularly relevant in a country such as Portugal, where, in contrast to other EU countries, ASc was first diagnosed in the absence of previous CSc cases.

## 4. Materials and Methods

### 4.1. Selection of Portuguese ASc Isolates

Among the first ASc cases, brainstem samples from two sheep to be AL_141_RQ/AF_141_RQ and scrapie resistant AL_141_RR/AL_141_RR genotypes, confirmed in 2004, were selected for this study. Cerebellum samples were not available as they were not routinely collected from sheep at that time. ASc was diagnosed after subjecting brainstem samples to rapid testing using approved lab tests and protocols as outlined (TeSeE™ kit, Bio-Rad, Munich, Germany) and to confirmatory tests histopathology, immunohistochemistry (anti-PrP 2G11 mouse antibody ovine PrP peptide sequence 146-R_154_-R_171_-182, Institute Pourquier, 1:200) and Western immunoblotting (TeSeE^®^ western blot kit, Bio-Rad) (see Table 1 for a summarized description of the isolates).

### 4.2. Transgenic Ovine ARQ PrP Mice (TgshpXI) Bioassay

All infection experiments in mice (LALLF 7221.3-2-1-027/02) described in this study were approved by the competent authority of the Federal State of Mecklenburg Western Pomerania, Germany, based on national and European legislation, namely the directive 2010/63/EU on the protection of animals used for scientific purposes.

Transmissions studies were conducted at the Friedrich–Loeffler–Institut (Isle of Riems, Germany). Each brainstem sample was prepared as a 10% homogenate in 0.9% sterile sodium chloride solution for intracerebral inoculation (30 µL). Samples from the two sheep genotypes were inoculated into transgenic TgshpXI mice overexpressing ovine ARQ genotype. Each genotype sample was replicated 15 times, resulting in a total of 30 animals. All mice were examined for clinical symptoms at least twice weekly. Incubation periods were determined as time between inoculation and death of animals. Mice were culled after manifesting clinical signs of disease, followed by removal of their brains for further examination. Animals dying less than 100 days after inoculation were excluded from the study.

### 4.3. Lesion Profile

Formalin-fixed (4% neutral buffered formalin), paraffin-embedded brains were sectioned coronally at the level of the medulla nuclei, midbrain, thalamus and basal ganglia. All sections (4 µm thick) were stained in hematoxylin and eosin (H&E), according to standard protocols (https://science.vla.gov.uk/tse-lab-net/, accessed on 20 January 2019). Lesion profiles were produced according to vacuolation severity in nine grey matter (GM) and three white matter (WM) regions. The vacuolation score ranged from 0 to 5 in GM regions and from 0 to 3 in WM regions, as previously described [21,22].

### 4.4. PrP^Sc^ Deposition

Immunohistochemistry was performed using monoclonal anti-PrP 2G11 mouse antibody (ovine PrP peptide sequence 146-R_154_-R_171_-182, Institute Pourquier, 1:100). Antigen retrieval was performed by immersion of dewaxed sections in formic acid (98%) for 5 min, then autoclaving in Citrate Buffer (10 mM pH = 6.12) at 121 °C for 30 min, followed by cooling in distilled water. Endogenous peroxidase was inhibited by treatment with 3% hydrogen peroxide in methanol for 30 min, then washed using tris saline buffer (pH = 7.6). After cover plate assemblage, sections were blocked with horse serum (20%) for 30 min to prevent non-specific binding. Primary anti-PrP 2G11 antibody (PrP Residues 146-R_154_-R_171_-182, Pourquier Institute) was applied at a dilution of 1/100 for one hour at room temperature, with immunodetection performed using biotinylated horse anti-mouse (dilution 1/200), avidin-biotin-peroxidase-complex and diaminobenzidine chromogen, as outlined (Vectastain Elite ABC Kit) and then counterstaining with Mayer’s Hematoxylin.

### 4.5. Western Immunoblot Analysis

Western immunoblot analysis was performed as described previously [23,24]. Briefly, 10% (*w*/*v*) brain homogenates were prepared in 0.42 mM sucrose solution containing 0.5% deoxycholic acid sodium salt (DOC) and 0.5% Nonidet P40 (NP 40). Gross cellular debris was removed by centrifugation (6000 rpm for 5 min) at room temperature followed by addition of Proteinase K (Boehringer Mannheim) to 200 μL of supernatant, to a final concentration of 50 μg/mL PK, then incubated at 55 °C for 1 h. Digestion was terminated by addition of 4 μL Pefabloc (Roche, Mannheim, Germany) and heating for 5 min at 95 °C. Digested homogenates were mixed with phosphotungstic acid (PTA) to a final concentration of 0.3% (*w*/*v*) PTA. Samples were incubated at 37 °C for 60 min with constant agitation before centrifugation at 13,300 rpm for 30 min at room temperature. The supernatant was carefully removed, pellets resuspended in sample buffer, heated for 5 min at 95 °C and loaded onto 16% Tris–polyacrylamide gels. Gels were transferred onto polyvinylidene fluoride membranes (Millipore, Burlington, MA, USA) and blocked for 1 h in 5% (*w*/*v*) non-fat milk powder in PBS containing 0.1% (*v*/*v*) Tween-20 (PBST). Membranes were incubated with L42 monoclonal antibody (recombinant ovine PrP residues 145–163, R-Biopharm, Darmstadt, Germany) at a concentration of 0.4 μg/mL for 1 h at room temperature before washing three times with PBST followed by incubation in a 0.15 μg/mL concentration of alkaline-phosphatase-conjugated anti-mouse immunoglobulin (Dianova) in PBST for 1 h at room temperature. Membranes were finally washed three times with PBST, and bound antibodies were detected using the chemiluminescent substrate CDP Star (Tropix) and visualized directly in an image analysis system (Versa Doc, Quantity One, Bio-Rad, Munich, Germany).

## Figures and Tables

**Table 1:** Atypical Scrapie cases selected for strain typing by mouse bioassay showing results of diagnostic observations and tests.

Sheep Genotype	Surveillance Stream	Bio-Rad TeSeE Kit Rapid Test	Histopathology	IHC	Western Immunoblotting
AL_141_RQ/ AF_141_RQ	Healthy slaughter	0.407 0.613 0.580 (positive cut-off 0.226)	Discrete vacuolation in the STN V	Globular and semi-globular immunolabelling in WM at the obex	Multiband electrophoretic profile 12–60 kDa
AL_141_RR/ AL_141_RR	Healthy slaughter	1.332 0.325 1.602 (positive cut-off 0.232)	Discrete vacuolation in the STN V	Globular and semi-globular immunolabelling in WM at the obex	Multiband electrophoretic profile 12–60 kDa

STN V, spinal tract nucleus of the trigeminal nerve; IHC, immunohistochemistry; WM, white matter.

## Data Availability

Data sharing is not applicable to this article.
